# Supplementary material for: Simulation as a Training Method for Electricity Workers’ Safety
Source: Int J Environ Res Public Health. 2021 Feb 8;18(4):1591. doi: 10.3390/ijerph18041591 (PMC7915987; doi:10.3390/ijerph18041591)
Supplement: Supplementary file 1 [file ijerph-18-01591-s001.pdf]

International Journal of
Environmental Research
and Public Health

MDPI

Supplementary

Table S1. The SPS Simulation checklist.

	**Task Management**		
1	Tool preparation	O	Does not prepare the tools before starting the task
		O	Prepares some of the tools in advance, but then, during the task, has to stop to get some other tools
		O	Prepares all the necessary tools and materials
2	Risk Assessment	O	Starts the activity before the necessary assessments (e.g., check of the metal parts, check of the integrity of the devices, check of all the items present in the scene)
		O	Starts the activity after checking only some of the items
		O	Starts the activity after all the necessary checks (e.g. checks the metal parts, checks the integrity of the devices, checks all the items present in the scene)
3	Personal Protective Equipment (PPE)	O	Not all the necessary items of the PPE are used
		O	Uses some of the PPE incorrectly (e.g., helmet visor up) or some of the PPE items are not necessary
		O	All the necessary items of the PPE are used
4	Work documentation (e.g., work planning, legal documents, etc.)	O	Is not read
		O	Is read only after having started the activity
		O	Is read before starting the activity and, if necessary, even after
5	Tools organization	O	Materials and tools are haphazardly stored, he does not find them because they are left around or hinder the activity
		O	Materials and tools are in place, but sometimes they are not at hand
		O	Materials and tools are stored orderly, effectively and at hand
6	Unexpected events management	O	Acts like there was no unexpected or anomalous situation (e.g., mismatching data, damages, tampering or particular characteristics of the environment)
		O	Changes the activity in order to manage the unexpected or anomalous situation
		O	Stops the activity and looks for further information (e.g., talks about it with the colleague or the operation centre, seeks for further details, etc.)

Relationship with the client

	(to fill only in case the client is present in the scenario)		
7	Information to the client	O	Does not provide the client any information about the activity
		O	Gives the client information about the activity only if requested or after having started the activity
		O	The client is informed about the activity in advance
8	Client management	O	Is not able to manage the client (e.g., has a conflict, the client doesn't follow his instructions, is distracted by client's behaviour)
		O	Is able to manage the client, but only after he hindered the activity or had an argument with him
		O	Makes sure that the client does not interfere during the activity (does not disturb, keeps him calm)
	**Teamwork** (to fill only in case two or more operators are present in the scenario)		
9	Coordination	O	Works without talking with the colleague about what to do
		O	Talks with the colleague only after having started the activity
		O	Starts the activity only after talking with the colleague about what to do
10	Situation assessment	O	Rarely talks with the colleague and the subject is not related to the current activity
		O	Discussing about the activity does not brings proposals, says "yes" to everything or does not share his opinions
		O	Discussing about the activity brings suggestions on how to operate, if he does not agree with the colleague, he explicitly shares his opinion
11	Listening to the colleague	O	When his colleague is speaking, he does not reply, nods, or he even talks over him
		O	When his colleague is speaking, sometimes he does not reply or even talks over him
		O	When his colleague is speaking, he gives him a feedback (explicitly confirms what he has just done or heard what the colleague said)
12	Requests to the colleague	O	He does not make requests to the colleague
		O	The requests are generic or ambiguous (e.g., "give me that thing")

		O	The requests are detailed and complete (e.g., "could you fetch me the screwdriver, which is in the toolbox, on that table?")
13	Updating the colleague	O	He does not update his colleague about what he's doing (which step of the activity)
		O	Updates his colleague only if explicitly requested
		O	Updates his colleague even if not explicitly requested
14	Management of risky behaviour and violations	O	His colleague does not have risky behaviours and violations
		O	Does not point out to his colleague his violations and risky behaviour
		O	He points out to his colleague his violations and risky behaviour
		O	Stops his colleague and points out his violations and risky behaviour

Table S2. The five scenarios outline

Scenario	Title	N° of workers	Task	Overall Learning Objective(s)	Physical Risk Type
1C	The Real Electricity Meter	1	Electricity Meter substitution	Potential risks management	Electric, Low-Dexterity Injuries
2C	The Construction Site	2	Temporary supply installation	Securing the work environment	Electric, Fall, Low-Dexterity Injuries
3C	Demolition	1	Supply cessation and Electricity Meter removal	Fraud identification	Electric, Low-Dexterity Injuries
4C	End Of Works	2	Temporary supply removal	SWP application	Electric, Fall, Low-Dexterity Injuries
5C	The New Three-phase	1(+1)	Three-phase Electricity Meter with three-phase Electricity Meter substitution	SWP application	Electric, Low-Dexterity Injuries

Complete Device List:

Training ground –

- 3 Manfrotto Trepied Befree MKBFRA4-BH
- 1 Zoom H6 portable Numeric recorder
- 1 YI 4K Action Camera Full HD 12MP WIFI 155° wide angle 4K/30 – black
- 1 Toshiba Exceria M302 microSDXC U3 (64Go, Class 10)

- 1 smartphone connected with the app Vysor
- 1 USB-B cable (20cm)
- 1 LIFE system 75.1HD4104 – 4/9CH containing 4 LIFE camera, the HUB system, and the corresponding cables
- 1 Switching power supply KD2054/12 (12Vdc – 5A)
- 1 Monitor
- 1 Laptop
- 3 ZEPHYR belts with the corresponding ZEPHYR sensors
- 1 SubZero SZW-50 Microphone system with 2 wireless microphone headsets
- 1 Behringer Micromix MX400 Line Mixer
- 1 jack cable 3.5mm
- 2 HDMI cable (20m)

Remote control room –

- 2 big screens (1 television, 1 projected)
- 1 beamer
- 1 sound system linked to the beamer

Table S3. An outline of the activities to be done before and after the simulation, in the simulation room and in the debriefing room.

SIMULATION ROOM	DEBRIEFING ROOM
	□ Read the description of the scenario<br>□ Choose the participant(s)<br>□ Give the Job Request Module to the participant(s)<br>□ Bring the participant(s) in the sim room
□ Set the chest-band sensor<br>□ Sit the participant on a chair (for 2min)<br>□ Set the microphones<br>□ Set the helmet with the action-cam	□ Assign observation task to every observer (who to observe)<br>□ Provide checklist
SIMULATION	
□ Sit the participant on a chair<br>□ Remove the microphone<br>□ Charge the microphone<br>□ Remove the helmet<br>□ Charge the action-cam<br>□ Remove the chest-bend sensor<br>□ Charge the chest-bend sensor	□ Fill in the checklist
Set the simulation room for the next scenario	DEBRIEFING

The Debriefing Checklist:

SPS checklist

- the participants to the simulation fill in the checklist about themselves
- the observers fill in the checklist about their colleagues, divided into two groups, facilitated by a trainer in the discussion of what they observed and what they would have done differently
- the participants go back to the debriefing room

- **Welcome back to the debriefing room:** the participants are greeted with an applause
- **Defusing**: the facilitator asks the participants "how do you feel?" in order to let them express possible feelings activated during the simulation (e.g., frustration, anger, embarrassment, etc.)

- **Analysis:** every participant describes what he had done. If the participants talk about the behaviour of his colleague, the facilitator will refocus the discussion on his own behaviour. Video recordings are used if necessary.
- **Reflection**: every participant says what he would like to change and what he this was appropriate of his performance. Video recordings are used if necessary.
- **Observers**: the two groups of observers share what they discussed filling in the SPS checklist.
- **Discussion:** The facilitator helps the participants and the observers to reflect on specific items. Video recordings are used if necessary.
- **Take home message**: every participant declares what has learned from this experience.

Table S4. The post training evaluation questionnaire.

**Thinking about the training course you attended today, we kindly ask you to rate your agreement to the following questions on a scale from 1 (not at all), to 5 (completely).**	**1**	**2**	**3**	**4**	**5**
Were the trainers able to create an open and non-judgmental environment?	□	□	□	□	□
How much did you feel engaged and stimulated by the course?	□	□	□	□	□
How much the scenarios represented situations that are similar to those you face in your everyday work activity?	□	□	□	□	□
How much realistic was the simulation?	□	□	□	□	□
Do you think the training course is useful for your job?	□	□	□	□	□
Would you like to attend similar training courses in the future?	□	□	□	□	□
How satisfied are you for having attended the course?	□	□	□	□	□

**How useful were the following activities? We kindly ask you to rate your agreement to the following questions on a scale from 1 (not at all), to 5 (completely).**	**1**	**2**	**3**	**4**	**5**
Usefulness of the first part of the training (introduction)	□	□	□	□	□
Usefulness of participating in simulated activities	□	□	□	□	□
Usefulness of watching simulated activities	□	□	□	□	□
Usefulness of discussion after simulated activities (debriefing)	□	□	□	□	□

Please, write here further comments and suggestions:

Table S5. Example of an evaluation sheet performed by a SME watching a simulated activity

Time	Phase	Description	Evaluation	Evaluation Explanation	1° operator	Client action	Notes
0'00	Analysis	Situation assessment	compliant		Job request module analysis		
0'35	Analysis	Situation assessment	compliant		Pushes button on EM		
0'42	Preparation		partially compliant	non-delimited working area	Takes the tools		
1'00	Preparation		partially compliant	shirt out of the pants	Buttons up jacket		
1'36	Preparation		partially compliant		Explains the problem to the client	Starts asking question about the intervention; violates the safety distance	Violation due to the lack of delimitation
1'56	Preparation		partially compliant		Asks the client to stay at a safe distance		
2'00	Deprogrammation		non-compliant		Starts deprogramming using the proper device		
2'14	Deprogrammation		non-compliant	forgot to perform the evaluation	1st isolation glove		He takes off the glove after few seconds
2'16	Deprogrammation		non-compliant		Asks the client to stay at safe distance		
2'56	Deprogrammation		non-compliant	forgot to perform the evaluation	1st isolation glove (again)		
3'24	Deprogrammation		non-compliant	forgot to perform the evaluation	Puts on both isolation gloves		
3'28	Deprogrammation		non-compliant		lowers the visor		
4'10	Work under electric voltage		non-compliant		starts unscrewing the EM		
5'38	Work under electric voltage		non-compliant	forgot to check the detector	uses the voltage detector		
13'19	Work under electric voltage		non-compliant		End of scenario		
